# Discordant neutralizing antibody and T cell responses in asymptomatic and mild SARS-CoV-2 infection

**DOI:** 10.1126/sciimmunol.abf3698

**Published:** 2020-12-23

**Authors:** Catherine J. Reynolds, Leo Swadling, Joseph M. Gibbons, Corinna Pade, Melanie P. Jensen, Mariana O. Diniz, Nathalie M. Schmidt, David K. Butler, Oliver E. Amin, Sasha N. L. Bailey, Sam M. Murray, Franziska P. Pieper, Stephen Taylor, Jessica Jones, Meleri Jones, Wing-Yiu Jason Lee, Joshua Rosenheim, Aneesh Chandran, George Joy, Cecilia Di Genova, Nigel Temperton, Jonathan Lambourne, Teresa Cutino-Moguel, Mervyn Andiapen, Marianna Fontana, Angelique Smit, Amanda Semper, Ben O’Brien, Benjamin Chain, Tim Brooks, Charlotte Manisty, Thomas Treibel, James C. Moon, COVIDsortium investigators, Mahdad Noursadeghi, COVIDsortium immune correlates network, Daniel M. Altmann, Mala K. Maini, Áine McKnight, Rosemary J. Boyton

**Affiliations:** 1 Department of Infectious Disease, Imperial College London, London, UK.; 2 Division of Infection and Immunity, University College London, London, UK.; 3 Blizard Institute, Barts and the London School of Medicine and Dentistry, Queen Mary University of London, London, UK.; 4 Barts Heart Centre, St Bartholomew's Hospital, Barts Health NHS Trust, London, UK.; 5 National Infection Service, Public Health England, Porton Down, UK.; 6 Wolfson Institute of Preventive Medicine, Barts and the London School of Medicine and Dentistry, Queen Mary University of London, London, UK.; 7 Viral Pseudotype Unit, Medway School of Pharmacy, Chatham Maritime, Kent, UK.; 8 Department of Infection, Barts Health NHS Trust, London, UK.; 9 Department of Virology, Barts Health NHS Trust, London, UK.; 10 Royal Free London NHS Foundation Trust, London, UK.; 11 William Harvey Research Institute, Barts and the London School of Medicine and Dentistry, Queen Mary University of London, London, UK.; 12 German Heart Centre and Charité University, Berlin, Germany.; 13 Institute of Cardiovascular Science, University College London, UK.; 14 Department of Immunology and Inflammation, Imperial College London, London, UK.; 15 Lung Division, Royal Brompton & Harefield NHS Foundation Trust, London, UK.

## Abstract

Understanding the nature of immunity following mild/asymptomatic infection with SARS-CoV-2 is crucial to controlling the pandemic. We analyzed T cell and neutralizing antibody responses in 136 healthcare workers (HCW) 16-18 weeks after United Kingdom lockdown, 76 of whom had mild/asymptomatic SARS-CoV-2 infection captured by serial sampling. Neutralizing antibodies (nAb) were present in 89% of previously infected HCW. T cell responses tended to be lower following asymptomatic infection than in those reporting case-definition symptoms of COVID-19, while nAb titers were maintained irrespective of symptoms. T cell and antibody responses were sometimes discordant. Eleven percent lacked nAb and had undetectable T cell responses to spike protein but had T cells reactive with other SARS-CoV-2 antigens. Our findings suggest that the majority of individuals with mild or asymptomatic SARS-CoV-2 infection carry nAb complemented by multispecific T cell responses at 16-18 weeks after mild or asymptomatic SARS-CoV-2 infection.

## Teaser

Uncoupling of neutralizing antibody and T cell responses to SARS-CoV-2 antigens occurs in some mild or asymptomatic cases of COVID-19.

## INTRODUCTION

Studies of adaptive immunity to SARS-CoV-2 include characterization of lethal, severe and mild cases (*1*–*8*). Understanding how long immunity lasts in people who have had mild or asymptomatic infection is crucial. Healthcare worker (HCW) cohorts exposed to and infected by SARS-CoV-2 during the early stages of the pandemic are an invaluable resource to study this question (*9*–*14*). The UK COVIDsortium is a longitudinal, London hospital HCW cohort, followed from the time of UK lockdown on 23^rd^ March 2020 (*9*, *10*); weekly nasopharyngeal swabs for SARS-CoV-2 polymerase chain reaction (PCR), serology and serum collection for antibody analysis and a self-reporting health questionnaire allowed capture of mild/asymptomatic infection around the time of onset, so duration of immunity could be tracked. The majority of healthy people infected in the community with SARS-CoV-2 have not been hospitalized and lack PCR confirmation of infection. A key public health concern is the extent to which immunity in mild or asymptomatic cases may confer protection from future infection (*6*, *15*–*18*). In this cohort 21.5% of the 731 HCW studied had laboratory confirmed infection and all were asymptomatic or had mild disease. We conducted a cross-sectional case-controlled sub-study (n=136) to analyze T cell and nAb immunity at 16-18 weeks after UK lockdown (table S1). We collected samples from 76 HCW with laboratory-defined evidence of SARS-CoV-2 infection and 60 HCW matched for age, gender and ethnicity that were consistently SARS-CoV-2 PCR negative and serology negative. Here, we set out to investigate whether asymptomatic or mild infection with SARS-CoV-2 confers specific nAb and T cell responses lasting to 16-18 weeks.

## RESULTS

### SARS-CoV-2 multispecific T cell response

A number of T cell studies investigating SARS-CoV-2 infection have described the presence of Th1 immunity (*7*)*.* We assessed SARS-CoV-2 T cell frequencies by IFNγ-ELISpot using three complementary approaches: whole protein (*1*), mapped epitope peptide (MEP) pools (*4*), and overlapping peptide (OLP) pools (*3*) (table S2). The use of whole protein allows assessment of CD4 T cell responses to naturally processed epitopes, whereas the MEP and OLP pools assessed a combination of CD4 and CD8 T cell responses directed against defined immunogenic regions and unbiased coverage of key viral proteins, respectively.

**Fig. 1:**
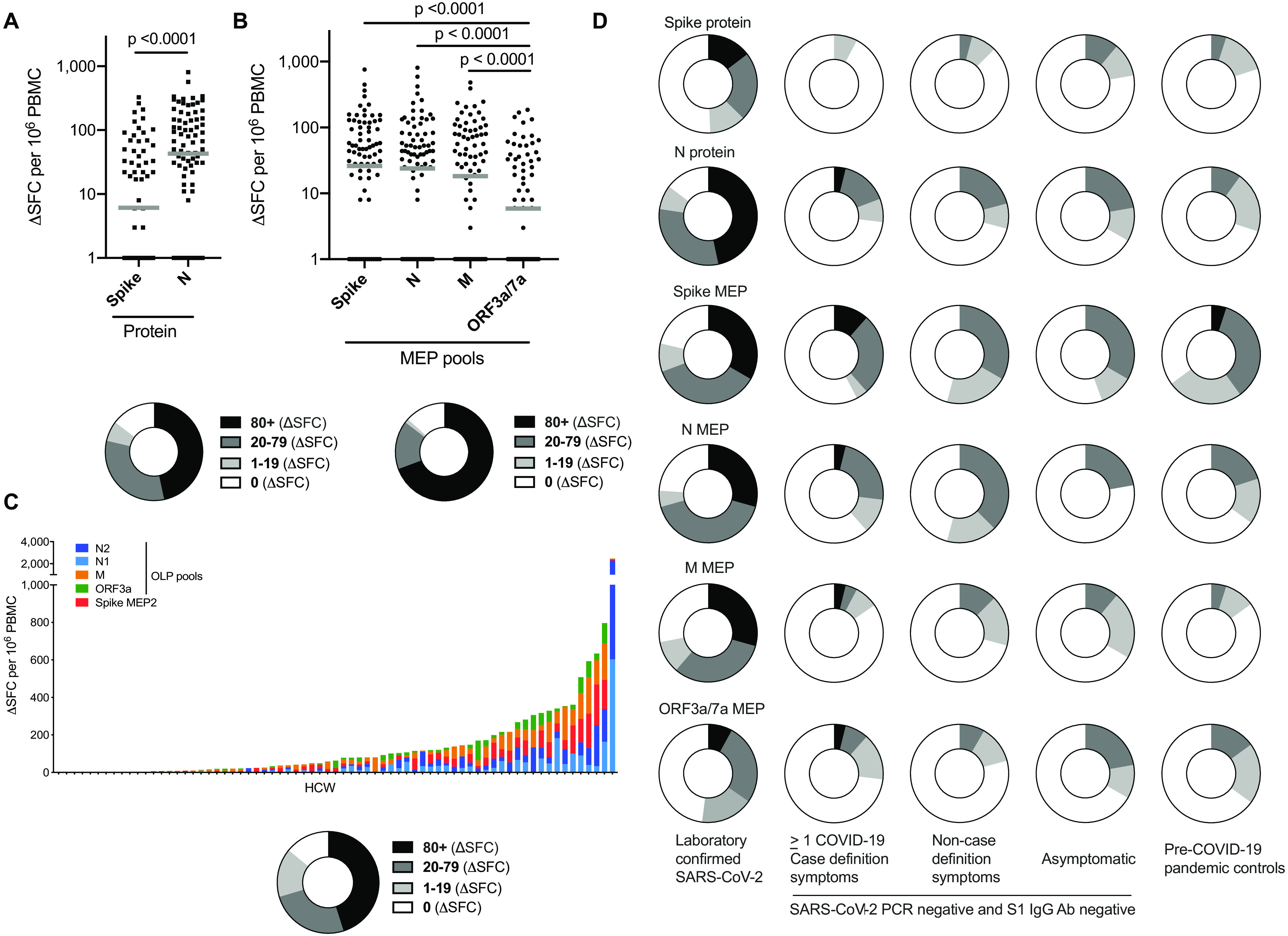
**T cell responses to SARS-CoV-2 antigens in HCW (laboratory-confirmed COVID-19) at 16-18 weeks after UK lockdown. (A-C)** Magnitude of T cell response and proportion of HCW with a summed T cell response within the given ranges (0, 1-19, 20-79, ≥80 ΔSFC/10^6^ PBMC). (**A**) Spike and N protein (n =75), (**B**) mapped epitope peptide (MEP; n = 75) and (**C**) overlapping peptide (OLP) pools (n =71, ordered by cumulative magnitude) in HCW with laboratory-confirmed SARS-CoV-2 infection (n = 75). (**D)** Proportion of HCW with a T cell response to SARS-CoV-2 individual proteins or peptide pools within given ranges (0, 1-19, 20-79, ≥80 ΔSFC/10^6^ PBMC) in the following groups: HCW cohort with laboratory-confirmed infection (n = 75); HCW cohort with no laboratory-confirmed infection but with one or more case-definition symptoms (n = 26), non-case-definition symptoms (n = 24) or asymptomatic (n = 9); pre-COVID-19 pandemic control cohort A (n = 20). (**A-B**) Bars at geomean. (**A**) Wilcoxon matched-pairs signed rank test. (**B**) Friedman multiple comparisons ANOVA with Dunn’s correction. Ab, antibody; HCW, health care workers; M, Membrane; ORF, open reading frame; N, nucleocapsid; S1, spike subunit 1; SFC, spot forming cells per 10^6^ PBMC.

Analyzing T cell responses to spike and nucleocapsid (N) stimulating with whole protein in HCW with mild or asymptomatic, laboratory confirmed infection, only 49% responded to spike whereas significantly more (85%) responded to N, showing a wide range of frequencies (Fig. 1A). Using MEP pools containing previously mapped immunogenic regions and offering good coverage for regional HLA genotypes (*4*), responses of >80 spot forming cells (SFC)/10^6^ peripheral blood mononuclear cells (PBMC) were found in 69% to peptide pools for spike, N, membrane (M) and open reading frame (ORF)3a/7a, with the latter being at a significantly lower frequency. Eighty-seven percent of HCW had detectable T cell responses to these MEP pools (Fig. 1B). A third T cell stimulation platform used OLP pools spanning the whole of N, M, and ORF3a, together with 15mers spanning immunogenic regions of spike (fig. S2B); using this approach, we assessed multi-specificity and cumulative SARS-CoV-2-specific T cell frequencies. This indicated a wide range of cumulative T cell response frequencies, from zero to >1000 SFC/10^6^ PBMC, with 89% showing a detectable T cell response (Fig. 1C). Of note with both the MEP and OLP platforms, responses to ORF3a/7a or ORF3a respectively were significantly lower than to other antigens (Fig. 1B; fig. S1A). Although T cell responses to individual regions were relatively weak, their cumulative frequencies across all pools tested were similar in magnitude to that of T cells directed against a pool of well-described CD8 epitopes from influenza, Epstein-Barr virus and cytomegalovirus (FEC), assessed in parallel in the same donors (fig. S1A), and comparable to frequencies found against SARS-CoV-1 pools following SARS infection (*19*).

Responses to spike, N whole protein and spike, N and M MEP were significantly higher frequency in HCW with laboratory confirmed SARS-CoV-2 infection than those in the matched group without laboratory evidence of infection (Fig. 1D, fig. S1, C-D). For example, 85% and 49% of HCW with laboratory confirmed SARS-CoV-2 had T cell responses to N and spike protein respectively, compared with 29% and 12% of SARS-CoV-2 PCR negative and S1 IgG negative HCW (p<0.0001) (Fig. 1D). T cell recognition of these stimuli in HCW without evidence of infection, irrespective of reported COVID-19-like symptoms, was similar to that seen in pre-COVID-19 pandemic controls (Fig. 1D, fig. S1C, D; table S1B). The OLP pools (utilizing increased cell numbers) showed detectable T cell responses in the PCR negative, S1 IgG negative HCW group (fig. S1B). With every T cell stimulation approach tested, responses were also seen in a proportion of pre-pandemic controls. Epitope mapping studies will be required to investigate possible cross-reactive components of these responses with other human coronaviruses as other studies have highlighted (*2*, *3*, *20*) and to assess the impact of any such cross-reactivity on disease outcome, whether positive or negative (*21*, *22*)*.*

In addition to IFNγ SFC, we explored other cytokines indicative of non-Th1 subset polarization by screening supernatants from spike and N protein-stimulated ELISpots; they showed no evidence of IL-4, 5, 13, 17 or 23 (fig. S1E). IL-2 release followed a similar pattern to that seen in the IFNγ ELISpots. However, TNFα was released by antigen-stimulated cultures in response to N across the cohort spectrum from those with laboratory confirmed infection to uninfected HCW and pre-pandemic controls, presumably reflecting an amplified response from other cell types including macrophages and NK cells.

In line with previous observations of SARS-CoV-2 T cells and aging (*23*), T cell responses in HCW (n=75) with laboratory confirmed SARS-CoV-2 correlated with age. There was a correlation with increasing age and T cell responses against spike MEP2, N1 OLP and ORF3a/7a MEP (fig. S2A-D). Broken down by age and gender, T cell immunity to spike increased with age in males (spike protein; r=0.522, p=0.006) (fig. S2E). We found no differences in T cell responses associated with ethnicity although the study was not sufficiently powered to report this negative finding (fig. S3A, B). T cell immunity to M MEP, ORF3a/7a MEP and ORF3a OLP was higher in males compared to females (fig. S3C, D).

### Neutralizing antibodies to SARS-CoV-2 at 16-18 weeks

**Fig. 2:**
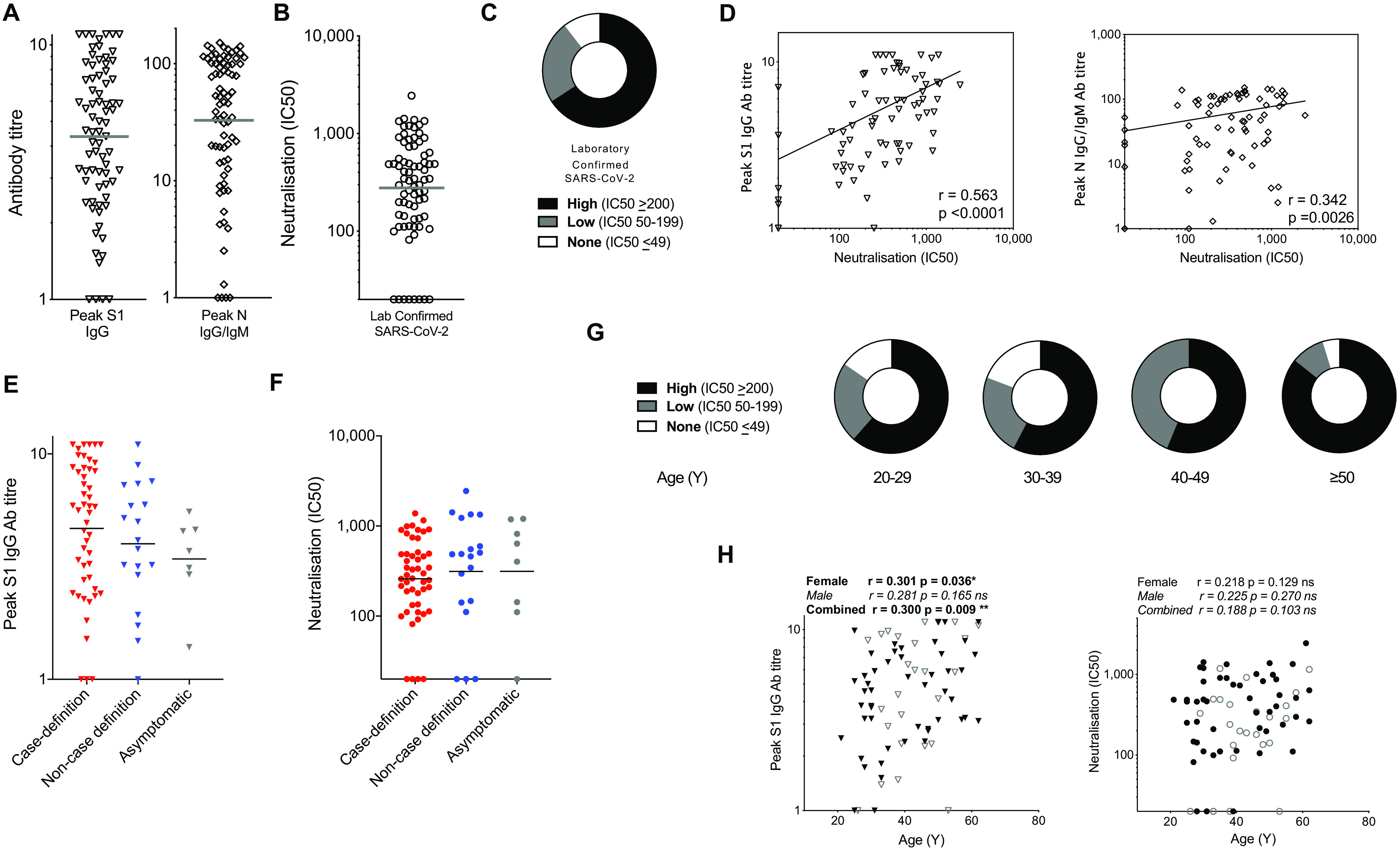
nAb responses to SARS-CoV-2 antigens in HCW (laboratory-confirmed COVID-19) at 16-18 weeks after UK lockdown. (**A**) Peak S1 IgG antibody titer and peak N IgG/IgM Ab titer across the study period in HCW with laboratory-confirmed SARS-CoV-2 infection (n = 76). (**B**) The distribution of nAb (IC50) titers across the cohort of HCW with laboratory-confirmed infection and (**C**) The proportion of HCW with an undetectable (0-49), low (50-199) or high (200+) nAb titer (IC50). (**D**) Correlation between peak S1 IgG Ab titer (left) or the peak N IgG/IgM Ab titer (right) and nAb titer (IC50) in HCW with laboratory-confirmed SARS-CoV-2 infection. (**E-F**) Peak S1 IgG Ab titer (**E**) and nAb titer (IC50) (**F**) in HCW with laboratory-confirmed infection, stratified by symptom group: ≥1 COVID-19 case-definition symptoms (Red), non-case definition symptoms (Blue) or asymptomatic (Grey) throughout trial and within 3-months of trial initiation. (**G**) The proportion of HCW with an undetectable (0-49), low (50-199) or high (200+) nAb titer (IC50) within specified age ranges; 20-29 years (n = 13), 30-39 years (n = 26), 40-49 years (n = 16) and ≥50 years (n = 21). (**H**) Correlations of age vs. peak S1 IgG Ab titer (left) and neutralizing antibody titer (IC50; right) in HCW with laboratory-confirmed SARS-CoV-2 infection separated by gender (female, black symbols; male, open symbols). (**D, H**) Spearman’s rank correlation, least squares log-log lines shown. (**A-B, E-F**) bars at geomean. (**E, F**) Kruskal Wallis multiple comparison ANOVA with Dunn’s correction, not significant. Ab, antibody; N, nucleocapsid; nAb, neutralizing antibody; S1, spike subunit 1; SFC, spot forming cells per 10^6^ PBMC; Y, years.

The majority of HCW in this cohort with laboratory confirmed SARS-CoV-2 infection had detectable S1 IgG and/or N IgG/IgM (97%) during follow-up; the longitudinal Ab response to S1 and N has been reported (*24*)*.* Peak antibody level during 16-18-week follow-up (Fig. 2A) was considered to be a useful marker of humoral immune activation in each HCW. There have been several reports relating cohort seroprevalence and Ab durability by S1, RBD or N ELISA to neutralizing antibodies and disease profile (*1*, *25*–*28*)*.* Some studies of nAb responses in severe, mild and asymptomatic disease have highlighted rapid waning of nAb within weeks (*14*–*16*, *29*), with others finding a more sustained neutralizing response (*26*, *30*, *31*). We analyzed the nAb response in HCW at 16-18 weeks after UK lockdown and found that 89% could neutralize pseudotyped virus. There was a range of nAb titers detectable, with 66% having an IC50 titer of >200 (Fig. 2B, C); as we discuss below, there is debate as to what IC50 indicates protection and we have cautiously benchmarked a correlate of protection by extrapolation to viral challenge in macaque studies (*32*). Eleven percent of HCW with laboratory-confirmed SARS-CoV-2 infection demonstrated no detectable neutralizing response (Fig. 2B, C). The eight HCW samples that tested negative by the pseudotyped virus neutralizing assay also tested negative by the authentic SARS-CoV-2 microneutralization assay. There was a positive correlation between IC50 measurements by the pseudotyped virus and authentic SARS-CoV-2 microneutralization assays (fig. S4A, B). Typical pseudotyped virus nAb profiles in the high (IC50 ≥200), low (IC50 50-199) and none (IC50 ≤49) categories are shown (fig. S5A). Note that the pseudotyped virus nAb response positively correlated with peak S1 IgG and peak N IgG/IgM, in line with other reported cohorts (1, 25-27) (Fig. 2D). Peak S1 IgG tended to be lower in those reporting non-case defining symptoms and those who were asymptomatic compared to those with case-definition symptoms (Fig. 2E). Importantly, the nAb IC50 titer at 16-18 weeks after lockdown was maintained at a similar level across these three symptom groups (Fig. 2F). Eighty-six percent of HCW aged ≥50y developed nAb at an IC50 of >200 compared with 58% of younger HCW aged 24-49y; p=0.0306 (Fig. 2G). Peak S1 IgG Ab increased with age in females (Fig. 2H). We looked in more detail at comparative features of infected individuals in the HCW cohort who did or did not show a nAb response at 16-18 weeks (fig. S5B-D); we cannot discount the possibility that these individuals may have shown an earlier response that had waned by 16-18 weeks. The 8 HCW with no nAbs spanned an age range of 26-53 years and tended to be at the lower end of the HCW age range. Although this sub-study was not powered to investigate stratified demographic differences, we looked at features such as gender, ethnicity, clinical role or location, use of personal protective equipment (PPE) or symptom profile and found no difference between those that made nAb and those that did not, though there was a trend to more male non-neutralizers.

### T cell and nAb responses are sometimes discordant

**Fig. 3:**
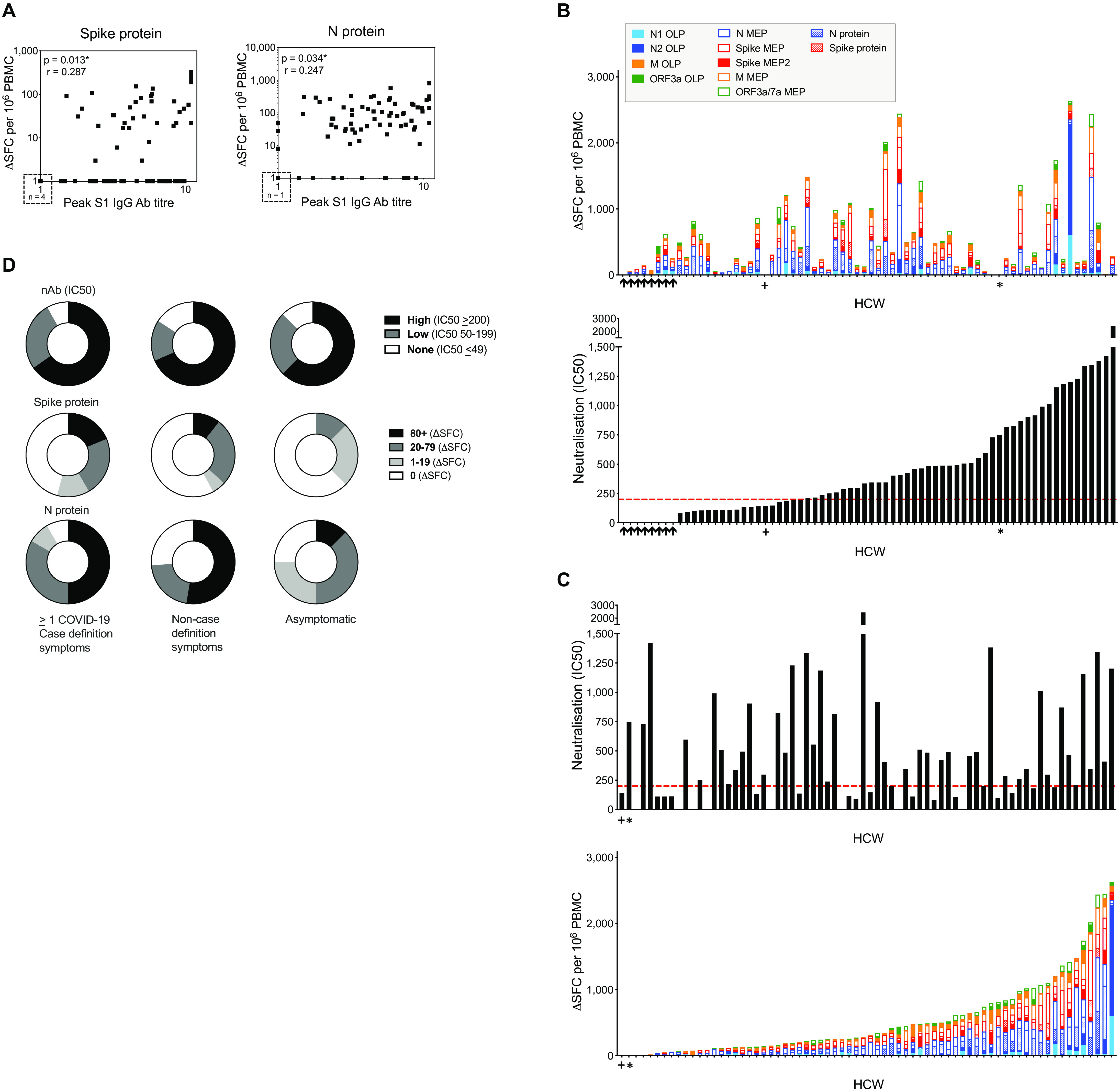
Concordant and discordant T cell and nAb responses in HCW (laboratory-confirmed COVID-19) at 16-18 weeks after UK lockdown. (**A**) Correlations between the peak S1 IgG Ab titer and T cell responses to spike protein (left) or N protein (right) in HCW with laboratory-confirmed SARS-CoV-2 infection (n = 75). (**B**) Top panel; Cumulative magnitude of the T cell response to spike and N proteins, mapped epitope peptide (MEP and MEP2) panels and overlapping peptide (OLP) panels (top panel) ordered by increasing magnitude of nAb response (bottom panel) or (**C**) Magnitude of nAb response (top panel) ordered by increasing cumulative magnitude of T cell response to spike and N proteins, MEP/MEP2 panels and OLP panels (bottom panel) in HCW with laboratory-confirmed SARS-CoV-2 infection (n = 70). HCW with no nAb (IC50 titer less than 50) are indicated by black arrows. + and * denote individuals with no T cell response to any protein or peptide pool. (**D**) Proportion of HCW with a nAb titer (IC50) or T cell response to spike and N proteins within given ranges stratified by symptom group; ≥1 COVID-19 case definition symptoms (n = 49 or 48), non-case definition symptoms (n = 19) or asymptomatic (n = 8) (**A**) Spearman’s rank correlation. HCW, health care workers; M, Membrane; ORF, open reading frame; N, nucleocapsid; nAb, neutralizing antibody; S1, spike subunit 1; SFC, spot forming cells per 10^6^ PBMC.

To better understand complementarity between nAb and T cells, we next compared the T cell, S1 IgG and nAb responses in individual HCW with laboratory confirmed SARS-CoV-2 (n=76). T cell responses to N and spike protein correlated with peak S1 IgG titer, but with weak correlation coefficients partly attributable to lack of T cell responses in some HCW with positive antibody titers to spike and N (Fig. 3A; blue box in fig. S6A, D). Correlations between peak N IgG/IgM titer and T cell responses to spike and N protein showed similar results (fig. S7A). Just over half of the HCW were discordant for T cell and S1 IgG responses, making no T cell response to spike protein, and 15% made no T cell response to N (fig. S6A-F). While we found no differences in terms of age, gender, ethnicity, symptom profile, clinical role or PPE use, there tended to be more non-responders among Black, Asian and Minority Ethnic (BAME) HCW. There was a correlation between the T cell response to spike protein (r=0.482, p<0.0001), spike MEP (r=0.412, p<0.0001) and peak S1 IgG titer across all the HCW studied (those with and without laboratory confirmed infection with SARS-CoV-2, n=133) (fig. S7B). T cell responses to spike protein (r=0.446, p<0.0001), spike MEP (r=0.343, p<0.0001) also correlated with nAb (IC50) (fig. S7B) but with some discordance.

In Fig. 2B, we showed that 11% of infected HCW lacked detectable nAb at 16-18 weeks after UK lockdown. To understand the complementarity between T cell and nAb responses in individual HCW, we analyzed responses of all HCW ranked either by nAb IC50 titer or cumulative T cell response. We first arrayed HCW responses ranked by magnitude of nAb response (Fig. 3B). Neutralization IC50 values for all HCW were plotted in relation to an indicative, protective cut-off value of >200 (dotted horizontal red line in lower panel). HCW lacking detectable nAb are indicated by 8 black arrows on the left. Their cumulative T cell response frequencies against viral antigens are shown in the panel above and are sometimes relatively low. Examining the converse, we then arrayed HCW responses ranked by magnitude of cumulative T cell response (Fig. 3C). From this plot, HCW with the lowest cumulative T cell response (to the left of the plot) have a range of nAb responses from none to >200 IC50. One young, asymptomatic, female HCW with a good peak S1 IgG titer had no T cell response to any antigens tested but made nAbs with a titer of 143 (Fig. 3B, C indicated by +). Another female HCW with a good S1 IgG titer, also had no T cell response to any antigens tested, but made nAbs with a titer of 747 (Fig. 3B, C indicated by *).

Of the 76 HCW studied with mild or asymptomatic laboratory-confirmed SARS-CoV-2 infection, 64% had one or more case-defining symptoms, 25% had non-case-defining symptoms and 11% were asymptomatic. Looking at T cell immunity and nAb levels across these symptom-stratified groups at 16-18 weeks, T cell responses tended to be higher in infected HCW with case defined symptoms. Responses to M MEP and ORF3a OLP were significantly higher in HCW reporting case definition symptoms than those that were asymptomatic (fig. S8A, B). Importantly, there was no significant fall in nAb titers across case-defining, non-case-defining symptoms and asymptomatic HCW groups (Fig. 3D) with 65%, 68% and 63% respectively showing an IC50 >200.

**Fig. 4:**
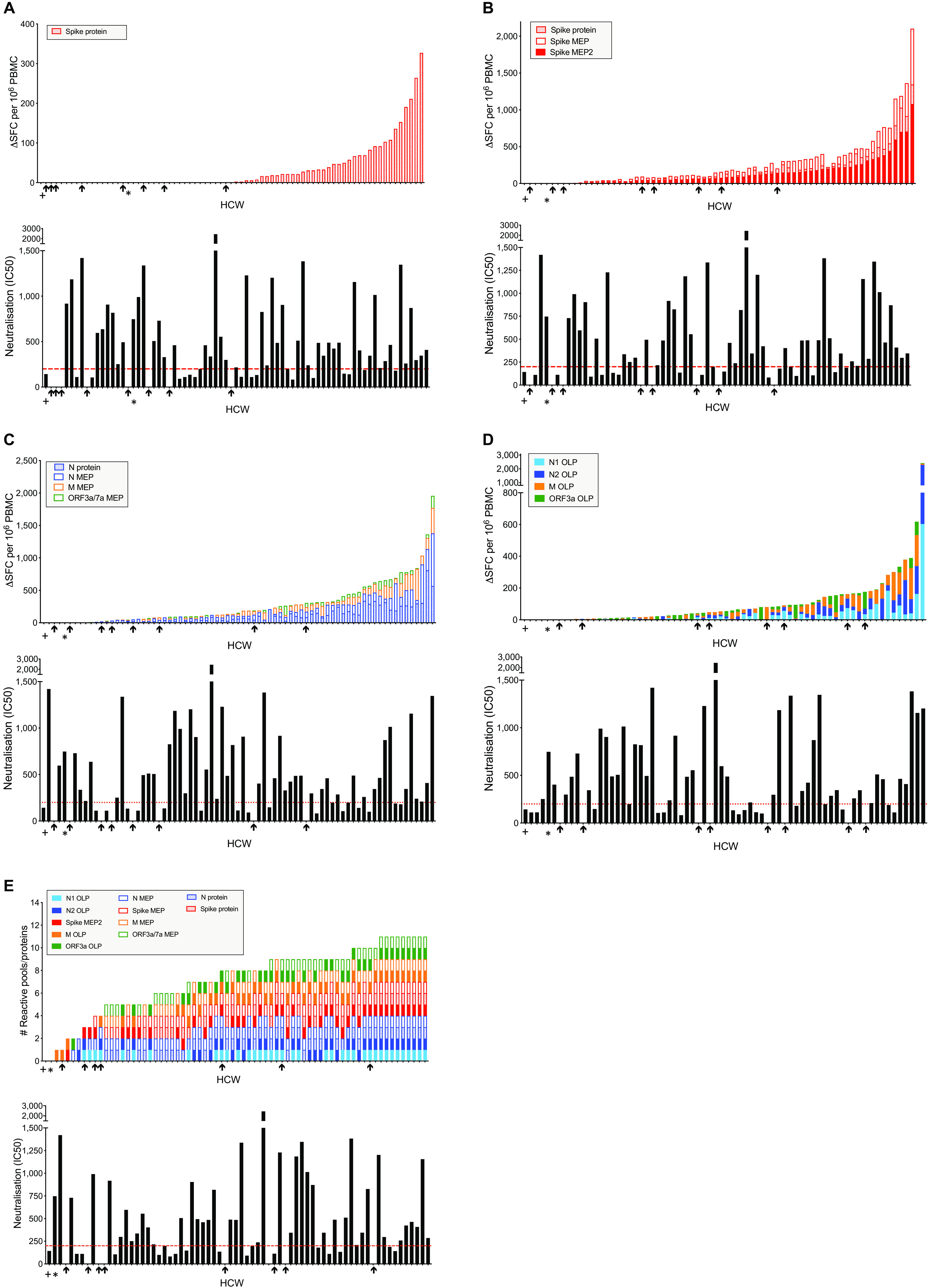
Discordant T cell and nAb responses broken down by T cell antigen. (**A-D**) Top panels; Magnitude of the T cell response to spike protein (n = 75) (**A**) Cumulative magnitude of T cell responses to spike protein and spike mapped epitope peptide (MEP and MEP2) pools (n = 70) (**B**) N protein and N, M and ORF3a/7a MEP pools (n = 75) (**C**) or N1, N2, M and ORF3a overlapping peptide (OLP) pools (n = 70) (**D**) ordered by increasing cumulative magnitude of T cell responses in HCW with laboratory-confirmed SARS-CoV-2 infection. Bottom panels; nAb titers (IC50) in HCW with laboratory-confirmed SARS-CoV-2 infection, ordered by corresponding top panel. (**E**) The number of reactive SARS-CoV-2 proteins or peptide pools (top panel) and nAb titer (IC50; bottom panel) in HCW with laboratory-confirmed SARS-CoV-2 infection (n = 70). Top panel ordered by cumulative magnitude; bottom panel ordered by top panel. HCW with no nAb (IC50 titer less than 50) are indicated by black arrows. + and * denote two individuals with no T cell response to any protein or peptide pool. HCW, healthcare workers; M, Membrane; N, nucleocapsid; nAb, neutralizing antibody; ORF, open reading frame; SFC, spot forming cells per 10^6^ PBMC.

Relating lack of nAbs to T cell responses to different specific antigens we show that none of the 8 HCW without detectable nAb make a T cell response to spike protein (Fig. 4A). The addition of data from spike MEP pools (potentially encompassing CD8 responses as well) revealed low T cell responses to spike in 5/8 HCW lacking a nAb response (Fig. 4B). Exploring T cell responses to N protein and N, M, and ORF3a/7a MEP pools showed 6/8 HCW without detectable nAb making a T cell response (Fig. 4C). Furthermore, there were OLP T cell responses in 6/8 HCW lacking nAb (Fig. 4D). Thus, HCW lacking nAb tend to lack responses to spike while maintaining low frequency T cells to other specificities. Assessing T cell responses ranked simply on the basis of presence or absence of recognition of proteins and peptide pools (rather than magnitude of response) indicates that those lacking a nAb response (black arrows) showed T cell responses against 1 to 5 antigens (Fig. 4E). Taken together, the data show discordance of nAb and T cell responses in individual HCW.

We looked in more detail at the data for some of the HCW showing discordant elements of adaptive immunity (fig. S9). There was a strong correlation between nAb IC50 and contemporaneous Euroimmun S1 IgG titer, yet thorough scrutiny of the plot also revealed a significant minority of individuals with discordant responses (fig. S9A). This discordance between S1 binding antibody titers and neutralization, may in part be due conformational differences between S1 protein in ELISA and virally expressed S protein or to presence of neutralizing antibodies targeting S2 (*33*, *34*). Two individuals with laboratory confirmed SARS-CoV-2 and a positive Euroimmun assay showed no detectable nAb response (fig. S9B, C). One of these showed no T cell response to spike, N protein or MEP, the other showed a modest T cell response. Interestingly, 12 HCW with laboratory confirmed SARS-CoV-2 showed a nAb response while also being negative for Euroimmun S1 IgG (fig. S9B, C). Four of the 12 HCW made cumulative T cell responses to spike and N protein in excess of 260 SFC/10^6^ and all made nAb responses of IC50 >99 and in the range 99 to 1184. This is an important finding since it showed that 12/76 (16%) HCW with proven infection with SARS-CoV-2 who had a negative Euroimmun test at about 4 months after infection still had potentially protective nAb and/or T cell responses. It also showed that 2/76 (3%) HCW with proven infection and a positive Euroimmun antibody binding test had no detectable nAb response. However, the majority of HCW with laboratory-confirmed infection did have a detectable (and likely protective) level of nAbs at 16-18 weeks. The 11% who lack detectable nAb have fewer T cells directed against spike but can show reactivity to other regions of the viral proteome.

## DISCUSSION

Much debate has focused on the possibility that the Ab response to SARS-CoV-2 may be short-lived, while T cell recognition may be strong, durable, and more common (*14*, *16*, *17*, *23*, *31*, *35*). Mild or asymptomatic infection are very common but are not usually diagnosed contemporaneously, making assessment of the durability of immunity in this common group challenging. Here we describe a cross-sectional case-controlled study of an exposed HCW cohort at 16-18 weeks after UK lockdown who had mild or asymptomatic infection picked up by repeated PCR and serological testing. This cohort shows variable T cell responses across the viral proteome sampled, with only two HCW with lab-confirmed COVID-19 showing no detectable T cell response across all the platforms tested. In this study, 89% of HCW with asymptomatic or mild COVID-19 had nAb at 16-18 weeks after UK lockdown and 66% had titers >200. In light of some reports of rapid waning of nAbs this result was surprising (*15*–*17*, *29*). A limitation of our study was that (like many others), we have here relied on pseudotyped virus neutralization as a surrogate for authentic virus neutralization. Our pseudotyped virus neutralization assay has been validated in this study with respect to authentic SARS-CoV-2 virus neutralization and previously (*36*). However, while pseudotyped virus neutralization is valuable and shows strong correlation with live virus neutralization, factors such as higher spike density on the wild-type virion may lead to nuanced differences in sensitivity (*37*). Here we show a complex pattern of T cell and nAb responses for individual HCW. Analysis of nAbs shows that the majority of laboratory confirmed SARS-CoV-2 infected HCW with no symptoms or only mild disease had relatively high nAb IC50 at 16-18 weeks after UK lockdown. In the absence of any proven human correlate of protection for SARS-CoV-2 infection, it is hard to be certain where to set the cut off for a ‘likely protective’ nAb IC50. The IC50s we measured were in the same range as those defined as conferring functional protection in macaque challenge studies, but this comparison has the caveat that high challenge doses were used in those studies (*32*). In terms of neutralization observations in humans, a study of SARS-CoV-2 susceptibility during an outbreak on a fishing vessel indicated a lack of infection in those showing a prior nAb titer (IC50) >1/160 (*38*). In infection by SARS-CoV-1, nAbs are often lost by 1-2 years after infection (*19*, *39*), whereas T cell responses can persist for up to 17y (*3*). Longitudinal follow-up of nAb versus T cell kinetics in the COVIDsortium cohort will illuminate T cell and nAb trajectories over time.

In terms of severe COVID-19 risk, two of the strongest factors identified have been gender and age (*40*). We found a positive correlation between peak S1 IgG Ab level and age in female study participants. Other observations have suggested a higher T cell response to mitogens in females with acute hospitalized COVID-19 (*41*); we observed higher memory T cell responses to spike antigen in older males. Thus, in this asymptomatic/mild cohort of HCW, the peak S1 IgG Ab level increases significantly with age in females, while it is the T cell response that increases significantly with age in males.

A limitation of our study is the fact that T cell and nAb responses were only measured at the 16-18-week cross-sectional time point. Ideally, T cell and nAb responses would be measured longitudinally to capture peak responses occurring in the period after infection and any differential decline in responses resulting from variations across individuals as seen with antibody binding responses (*24*). Another caveat is the fact that due to limitations of blood sample volume we only used one detection system to measure T cell responses, the IFNγ ELISpot. Other labs have opted to elicit low frequency responses by prior expansion in peptides with IL-2. We considered that such expansion might obviate our ability to draw direct conclusions about response frequencies.

A cautionary note about the ephemeral nature of adaptive immunity to coronaviruses comes from data for annual reinfections with the four seasonal coronaviruses and emerging data for reinfection by SARS-CoV-2 (*42*, *43*). Some studies have raised concern about the durability of serum antibodies and B cell memory, with data pointing toward impaired germinal center reactions in severe acute COVID-19 (*35*). Other studies have focused on the potential for rapid waning of nAb after mild SARS-CoV-2 infection (*14*, *15*). However, we find nAb detectable in the majority of HCW sampled 16-18 weeks after mild/asymptomatic infection. Some T cell data indicates that even asymptomatic people and household contacts develop low-frequency T cell responses, in line with results from the HCW without laboratory confirmed infection using one of our platforms with higher T cell numbers (*6*). We show here that infected HCW can display highly heterogeneous T cell recognition of epitopes from diverse SARS-CoV-2 structural and non-structural proteins, but it is not yet possible to decode the differential impacts of these responses for protection. Analysis of T cell response repertoire in convalescent, hospitalized COVID-19 patients argues that breadth of T cell response is a marker of mild disease (*44*).

While Ab and/or T cell data have been reported in several settings (*1*–*8*, *13*–*16*, *23*–*31*), many studies lack the granularity to relate binding Ab, nAb, and broad-range T cell response analysis to long-term immunity after asymptomatic or mild disease – the common COVID-19 experience of the majority of individuals. Our cohort study highlights the heterogeneity of immune memory in exposed individuals with mild or asymptomatic infection, cautioning against simple assertions about ‘typical’ responses in most people. The cohort shows discordance between nAb and T cell responses with some individuals showing good nAb responses alongside low T cell responses and vice versa. When T cell responses are present, they are of variable frequency and specificity. There are also HCW lacking evidence of seroreactivity by S1 Euroimmun assay, yet with evidence of a positive neutralizing antibody response. While we find relative discordance of responses in our study comprising individuals at 4 months after infection, other reports of concordance either between S1 Ab and neutralization or between neutralization and spike CD4 T cell responses tended to analyze within the first weeks after infection (*2*, *45*).

In summary, we find that in the majority of these working adults there is immunity at 16-18 weeks comprising nAb (often at a level likely to protect), usually complemented by multi-specific T cell responses. Understanding protective immunity in the population will require simultaneous scrutiny of T cell and antibody responses.

## MATERIALS AND METHODS

### Study design

**Fig. 5:**
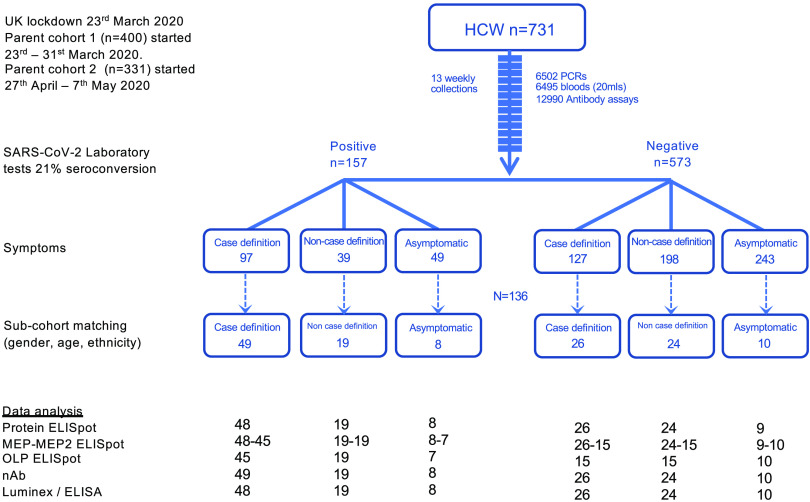
CONSORT flow diagram for the COVIDsortium London healthcare worker cohort and sub-cohort. CONSORT flow diagram showing participant recruitment into COVIDsortium London healthcare worker parent study and sub-study. Participants were stratified by SARS-CoV-2 PCR and antibody laboratory tests and by symptoms experienced during 16 weeks follow-up and in the 3 months prior to study initiation. SARS-CoV-2 laboratory test positive and negative participant sub-cohort groups were matched for gender, age and ethnicity.

We conducted a cross-sectional case-controlled sub-study of 136 hospital based HCW at 16-18 weeks after UK lockdown (Fig. 5). Seventy-six HCW with mild/asymptomatic laboratory confirmed SARS-CoV-2 infection captured by weekly SARS-CoV-2 PCR and Euroimmun/Roche antibody tests were recruited. An age, sex, symptom and ethnically matched group of 60 HCW with similar exposure that remained SARS-CoV-2 PCR negative and Euroimmun/Roche antibody test negative throughout the 16-week follow up period were also recruited. The HCW completed a symptom diary and were divided into those that reported one or more case definition symptoms, non-case definition symptoms or were asymptomatic during the 16-week follow up period and in the 3 months prior to the start of the study. The main objective of the study was to investigate T cell and neutralizing antibody immunity to SARS-CoV-2 infection in asymptomatic/mild COVID-19 in a working adult cohort.

### Ethics statement

The COVIDsortium Healthcare Workers bioresource was approved by the ethical committee of UK National Research Ethics Service (20/SC/0149) and registered on ClinicalTrials.gov (NCT04318314). The study conformed to the principles of the Helsinki Declaration, and all subjects gave written informed consent.

Pre-pandemic healthy donor samples were collected and cryopreserved before October 2019 (table S2). Pre-pandemic cohort A and B samples were recruited under ethics numbers 17/LO/0800 and 11/LO/0421 respectively.

### COVIDsortium Healthcare Worker Participants

Adult HCW (>18 years old) from a range of clinical settings who self-declared as fit to attend work were invited to participate via local advertisement of the project (see https://covid-consortium.com). Full study details of the bioresource (participant screening, study design, sample collection, and sample processing) have been previously published (*10*).

A cohort of 400 HCW was initially recruited from St Bartholomew’s Hospital, London, in the week of UK lockdown (23^rd^-31^st^ March 2020). All participants were asymptomatic and self-declared fit to attend work in hospital. Recruitment was extended (27^th^ April-7^th^ May 2020) to include 331 additional participants from multiple sites: St Bartholomew’s Hospital (n=101 additional), NHS Nightingale Hospital (n=10), and Royal Free NHS Hospital Trust (n=220).

A prospective, observational, longitudinal cohort design was used and consisted of questionnaires exploring demographic, clinical and exposure risks, and sample collection at baseline and weekly follow-up for 15w from the start of each cohort. Participants were asked to provide details and timing of symptoms in the 3 months prior to baseline, and for those who were unable to attend follow-up visits (due to shift rostering, annual leave or self-isolation), the reason for non-attendance was collected, to ensure capture of information regarding isolation due to participant symptoms or household contacts. On return from self-isolation with symptoms, convalescent samples were collected. Further follow-ups at 6 and 12 months are planned.

Complete details of the sampling protocol have been previously published (*10*). Initial analysis of samples for determining infection with SARS-CoV-2 included: nasal RNA stabilizing swabs baseline and weekly with reverse transcriptase polymerase chain reaction (RT-PCR): Roche cobas® SARS-CoV-2 test; Ab testing baseline and weekly: IgG Ab assay to spike protein S1 antigen, (EUROIMMUN Anti-SARS-CoV-2 enzyme-linked immunosorbent assay [ELISA]); and anti-nucleocapsid total antibody assay (ROCHE Elecsys Anti-SARS-CoV-2 electrochemiluminescence immunoassay [ECLIA]). Antibody ratios > 1.1 were considered test positive for the EUROIMMUN SARS-CoV-2 ELISA and >1 was considered test positive for the ROCHE Elecsys anti-SARS-CoV-2 ECLIA following published Public Health England (PHE) evaluations (*46*, *47*).

At baseline, information relating to demographics and exposures was collected via a standardized questionnaire. Mean age of the cohort (n=731) was 38 ± 11 years; 33% are male, 31% nurses, 20% doctors, and 19% work in intensive care units. COVID-19-associated risk factors were: 37% Black, Asian or minority ethnicities (BAME); 18% smokers; 13% obesity; 11% asthma; 7% hypertension and 2% diabetes mellitus (*10*). At weekly follow-up visits information relating to symptom burden was recorded using a standardized questionnaire. Symptoms were classified as follows: ‘case-defining’ (fever, new continuous dry cough or a new loss of taste or smell), ‘non-case-defining’ (specific symptoms other than case-defining symptoms, or unspecified symptoms), or asymptomatic (no symptoms reported).

Case definition for coronavirus disease 2019 (COVID-19), as of 29 May 2020 European Centre for Disease Prevention and Control [https://www.ecdc.europa.eu/en/covid-19/surveillance/case-definition]

A total of 731 HCW underwent 16 weeks of serial assessment (attending unless ill, self-isolating, on holiday, or redeployed). Across the main study cohort, 48 participants had positive RT-PCR results with 157 (21.5%) seropositive participants. Infections were asymptomatic or mild with only two hospital admissions (neither requiring intensive care admission, both discharged well). The cohort therefore represents working age community COVID-19 rather than hospitalized COVID-19.

In London, the case-doubling time in March, 2020 was approximately 3–4 days. The number of nasal swabs testing positive for SARS-CoV-2 in our study peaked at March 23^rd^ to 31st, 2020 suggesting that infections peaked on or around March 23^rd^, 2020, the day of UK lockdown. We thus observed approximately synchronous infections coincident with the peak epidemic transmission in London at the start of the study, UK lockdown on March 23^rd^ and therefore used this as the benchmark starting point for our analysis of T cell and nAb responses in the first wave (*24*).

The cross-sectional case controlled sub-study (n=136) collected samples at 16-18 weeks after UK lockdown (Table S1, Fig. 5). The cross-sectional case controlled sub-study included 76 HCW (mean age 41y, 36% male) with laboratory defined evidence of SARS-CoV-2 either by SARS-CoV-2 positive PCR and/or positive for spike IgG (Euroimmun ELISA)/ N IgG/IgM antibody (Roche Elecsys). Fifty-seven percent reported one or more case defining COVID-19 symptoms. Twenty-four percent reported non-case defining symptoms and 19% were asymptomatic at baseline, during 16-week follow-up or in the 3 months prior to baseline. A second age, gender, and ethnicity matched subgroup of sixty HCW were recruited (mean age 39y, 37% male) who were SARS-CoV-2 PCR negative and negative for spike IgG (Euroimmun ELISA) and N IgG/IgM antibody (Roche Elecsys) tests throughout the 16-week follow-up. However, forty-four percent reported one or more case defining COVID-19 symptoms, 41% non-case defining symptoms and 15% were asymptomatic at baseline, during 16-week follow-up and in the 3 months prior to baseline. There was no significant difference in T cell or nAb responses measured in HCW with laboratory confirmed SARS-CoV-2 that were recruited into the parent study via cohort 1 or 2 (fig. S10).

### Isolation of PBMC

Peripheral blood mononuclear cells (PBMC) were isolated from heparinized blood samples using Pancoll (Pan Biotech) or Histopaque®-1077 Hybri-Max^TM^ (Sigma-Aldrich) density gradient centrifugation in SepMate tubes (StemCell) according to the manufacturer’s specifications. Isolated PBMCs were cryopreserved in fetal calf serum containing 10% DMSO and stored in liquid nitrogen.

### Isolation of serum

Whole blood samples were collected in SST vacutainers (VACUETTE® #455092) with inert polymer gel for serum separation and clot activator coating. After centrifugation at 1000 X g for 10 min at room temperature, serum layer was aliquoted and stored at -80^0^C for specific SARS-CoV-2 Ab titer detection by ELISA and for SARS-CoV-2 spike pseudotyped virus neutralization assays.

### SARS-CoV-2 specific Ab titer

SARS-CoV-2 antibody testing was carried out in the laboratories of Public Health England UK using two commercial assays following the manufacturers’ instructions. The Euroimmun anti-SARS-CoV-2 enzyme-linked immunosorbent assay (ELISA) (IgG) measures serum IgG against SARS-CoV-1 S1 antigen (*46*) and the Roche Elecsys Anti-SARS-CoV-2 electrochemiluminescence immunoassay (ECLIA) measures serum antibody (including IgG) directed against the SARS-CoV-2 N (*47*). The Euroimmun ELISA was carried out using a Stratec Gemini automated microplate processor. Raw optical density OD 450nm readings were adjusted by calculating the ratio of the OD of control or participant sample divided by the calibrator OD. A ratio of ≥ 1.1 was deemed positive. A ratio of 11 was taken to be the upper threshold as the assay saturates beyond this point. The Roche ECLIA was performed using a Roche Cobas^®^ e801 Immunoassay Analyzer. Results were expressed as a cut-off index (COI) calculated by the analyzer software as the electrochemiluminescence signal obtained from the participant sample divided by the lot-specific cut-off value. A COI value ≥ 1 was deemed positive. Across their dynamic range, the semiquantitative indices of both assays approximate to a linear relationship with antibody level (*24*).

### Recombinant proteins

The SARS-CoV-2 S1 spike antigen and nucleocapsid proteins were obtained from the Centre for AIDS Reagents (CFAR), National Institute for Biological Standards and Control (NIBSC), UK and consisted of SARS-CoV-2 nucleoprotein and S1 spike antigen from Dr. Peter Cherepanov, Francis Crick Institute, UK.

### Mapped epitope pools (MEP)

Pools of 13-20mer peptides based on the protein sequences of SARS-CoV-2 S1 (spike), nucleocapsid (N), membrane (M) and open reading frames 3a and 7a (ORF3a/7a) described previously were synthesized (*4*) (GL Biochem Shanghai Ltd, China). To stimulate PBMC, separate pools of sequences for Spike (18 peptides), N (10 peptides), M (6 peptides) and ORF3a/7a (7 peptides) were used (table S2). A second mapped epitope pool of SARS-CoV-2 S1 peptides (spike MEP2) based on alignment of all sequences of published SARS-CoV-1 epitopes (www.iedb.org; search criteria: positive assays only, T cells assays, host: human) with the spike-SARS-CoV-2 sequence and 15-mer peptides synthesized to cover the homologous sequences. In addition, we synthesized 15-mer peptides covering the predicted SARS-CoV-2 spike epitopes (*3*) to give a total of 55 peptides in this pool (Spike MEP2) (table S2)

### Overlapping peptide pools (OLP)

15-mer peptides overlapping by 10 amino acids spanning the entire protein sequence of SARS-CoV-2 nucleocapsid (N), Membrane (M) and ORF3a were synthesized (GL Biochem Shanghai Ltd) (Table 2). To stimulate PBMC, the peptides were divided into 4 pools covering N (N1, N2, 41 peptides each), M (43 peptides), and ORF3a (53 peptides).

### IFNγ-ELISpot Assay

Unless otherwise stated, culture medium for human T cells was sterile 0.22μM filtered RPMI medium (GibcoBRL) supplemented with 10% by volume heat inactivated (1h, 64°C) fetal calf serum (FCS; Hyclone, and 1% by volume 100x penicillin and streptomycin solution (GibcoBRL).

For experiments involving T cell stimulation with proteins or MEP peptide pools, pre-coated ELISpot plates (Mabtech 3420-2APT) were washed x4 with sterile PBS and were blocked with R10 for 1h at room temperature. 200,000 PBMC were seeded in R10/well and were stimulated for 18-22h at 37°C with 5%CO_2_ with SARS-CoV-2 recombinant proteins (10μg/ml) or MEP pools (10μg/ml/peptide). Internal plate controls were R10 alone (without cells) and anti-CD3 (Mabtech mAb CD3-2). At the end of the stimulation period, cell culture supernatants were collected and stored for later cytokine analysis by Luminex and ELISA. ELISpot plates were developed with human biotinylated IFNγ detection Ab, directly conjugated to alkaline phosphatase (7-B6-1-ALP, Mabtech; 1μg/ml), diluted in PBS with 0.5% FCS, incubating 100μl/well for 2h at room temperature. This was followed by 100μl/well of sterile filtered BCIP/NBT-plus Phosphatase Substrate (Mabtech) for 5 min at room temperature. Plates were washed in ddH20 and left to dry completely before being read on AID-ELISpot plate reader. For experiments involving T cell stimulation with OLP peptide pools and spike MEP2 pool ELISpot plates (Merck-Millipore, MSIP4510) were coated with human anti-IFNγ Ab (1-D1K, Mabtech; 10μg/ml) in PBS overnight at 4°C. Plates were washed x6 with sterile PBS and were blocked with R10 for 2h at 37°C with 5% CO_2_. PBMC were thawed and rested in R10 for 3h at 37°C with 5% CO_2_ before being counted. 400,000 PBMC were seeded in R10/well and were stimulated for 16-20h with SARS-CoV-2 OLP pools or spike MEP2 pool (2μg/ml/peptide). Internal plate controls were R10 alone (without cells) and two DMSO wells (negative controls), concanavalin A (ConA, positive control; Sigma-Aldrich) and FEC (HLAI-restricted peptides from influenza, Epstein-Barr virus, and CMV; 1μg/ml/peptide). ELISpot plates were developed with human biotinylated IFN-γ detection antibody (7-B6-1, Mabtech; 1μg/ml) for 3h at room temperature, followed by incubation with goat anti-biotin alkaline phosphatase (Vector Laboratories; 1:1000) for 2h at room temperature, both diluted in PBS with 0.5% BSA by volume (Sigma-Aldrich), and finally with 50μl/well of sterile filtered BCIP/NBT Phosphatase Substrate (ThermoFisher) for 7 min at room temperature. Plates were washed in ddH20 and left to dry overnight before being read on an AID classic ELISpot plate reader (Autoimmun Diagnostika GMBH, Germany).

Analysis of ELISpot data was performed in Microsoft Excel. The average of two R10 alone wells or DMSO (Sigma-Aldrich) wells was subtracted from all peptide stimulated wells and any response that was lower in magnitude than 2 standard deviations of the sample specific control wells was not considered a peptide specific response. Results were expressed as difference in (delta) spot forming cells per 10^6^ PBMC between the negative control and protein/peptide stimulation conditions. We excluded the results if negative control wells had >100 SFU/10^6^ PBMC or positive control wells (ConA or anti-CD3) were negative. Results were plotted using Prism v. 7.0e and 8.0 for Mac OS (GraphPad).

### Cytokine measurement

Concentrations of IL-2 and TNF𝛼 in cell culture supernatants in response to PBMC stimulation with spike or N protein were measured by ELISA using Duo-set® antibody pairs and standards (Bio-Techne). Optical density measurements were performed on a FLUOstar Omega Microplate reader (BMG Labtech). Concentrations of IL-4, IL-5, IL-13, IL-17a and IL-23 were measured by multiplex Luminex® assay (Bio-Techne) on a Bio-Plex 200 instrument (Bio-Rad Laboratories, Ltd). Cytokine levels were calculated in Microsoft Excel and concentrations for protein stimulated samples obtained by subtracting values for media only controls. Standard curves were plotted using Prism 8.0 for Mac OS (GraphPad).

### Cell Lines

HEK-293T and Huh7 (both ATCC) were cultured and maintained in high glucose Dulbecco’s Modified Eagle’s Medium and supplemented with GlutaMAX, 10% (*v/v*) heat-inactivated fetal bovine serum (FBS, 56°C for 30 min), 100IU/ml penicillin and 100μg/ml streptomycin. Cell lines were cultured at 37°C with 5% CO_2_.

### Production and titration of SARS-CoV-2 pseudotyped lentiviral reporter particles

Pseudotype stocks were prepared by linear polyethylenimine 25K (Polysciences) co-transfection of HEK-293T (ATCC) with SARS-CoV-2 spike pcDNA expression plasmid, HIV gag-pol p8.91 plasmid and firefly luciferase expressing plasmid pCSFLW at a 1:1:1.5 ratio (*47*, *48*). 2.5x10^4^ cells/cm^2^ were plated 24h prior to transfection in 60cm^2^ cell culture dishes. 48 and 72h post transfection, pseudotype-containing culture medium was harvested and centrifuged at 500xg for 5 min to clear cell debris. Aliquots were stored at -80°C. TCID assays were performed by transduction of Huh7 cells to calculate the viral titer and infectious dose for neutralization assays. p24 ELISA was also used to determine input concentration.

### p24 ELISA

Pseudotype stock concentrations were determined by ELISA for p24 protein concentration as previously described (*49*). White ELISA plates were pre-coated with 5μg/ml sheep anti-HIV-1 p24 antibody (Aalto Bio Reagents) at 4°C overnight. Pseudoviral supernatants were treated with 1% Empigen BB (Merck) for 30 min at 56°C and then plated at 1:10 dilution in Tris-buffered saline (TBS) on pre-coated plates and incubated for 3h at room temperature. Alkaline phosphatase-conjugated mouse anti-HIV-1 p24 monoclonal antibody (Aalto Bio Reagents) in TBS, 20% (*v/v*) sheep serum, 0.05% (*v/v*) Tween 20 was then added and incubated for 1h at room temperature. After 4 washes with phosphate-buffered saline (PBS)-0.01% (*v/v*) Tween 20 and 2 washes with ELISA Light washing buffer (ThermoFisher), CSPD substrate with Sapphire II enhancer (ThermoFisher) was added and incubated for 30 min at room temperature before chemiluminescence detection using a CLARIOStar Plate Reader (BMG Labtech).

### Pseudotyped SARS-CoV-2 neutralization assays

SARS-CoV-2 pseudotype neutralization assays were conducted using pseudotyped lentiviral particles as previously described (*48*–*51*). The pseudotype virus assay used here was developed, characterized and validated relative to live virus by ourselves and one of the authors previously (36). Serum was heat-inactivated at 56°C for 30 min to remove complement activity. Serum dilutions in DMEM were performed in duplicate in white, flat-bottom 96-well plates (ThermoFisher, #136101) with a starting dilution of 1 in 20 and 7 consecutive 2-fold dilutions to a final dilution of 1/2,560 in a total volume of 100μl. 1 x10^5^ RLU of SARS-CoV-2 pseudotyped lentiviral particles were added to each well and incubated at 37°C for 1h. 8 control wells per plate received pseudotype and cells only (virus control) and another 8 wells received cells only (background control). Negative controls of pooled pre-pandemic sera, collected prior to 2008, and a positive neutralizer were spaced throughout the plates. RLUs for each well were standardized against technical positive (virus control) and negative (cells only) controls on each plate to determine a percentage neutralization value. 4x10^4^ Huh7 cells suspended in 100μl complete media were added per well and incubated for 72h at 37°C and 5% CO_2_. Firefly luciferase activity (luminescence) was measured using Steady-Glo® Luciferase Assay System (Promega) and a CLARIOStar Plate Reader (BMG Labtech). An average neutralization was calculated across two sample replicates for each serum dilution. Neutralization curves for each serum sample were plotted and the percentage neutralization modeled as a logistic function of the serum dilution factor (log10). Representative neutralization curves for ‘High’, ‘Low’ and ‘None’ neutralizers are shown in Fig. S5A. A non-linear regression (curve fit) method was used to determine the dilution fold that neutralized 50% (IC50). A result of IC50 greater than 49 was deemed positive.

### Authentic SARS-CoV-2 and titration

SARS-CoV‐2 strain *2019‐nCoV*/*BavPat1/2020* authentic virus cell culture supernatant (isolate collection date 1^st^ January 2020) was purchased from the European Virus Archive Global (EVAg, Charité Universitätsmedizin Berlin, Germany). VeroE6 were seeded in 75cm^2^ cell culture flasks 24h before inoculation with virus cell culture supernatant containing 2.2 × 10^6^ PFU in a volume of 10ml DMEM 10% FBS. Flasks were observed daily and virus-containing cell culture medium was harvested when >80% of cells showed cytopathic effect (CPE). Supernatant was centrifuged at 500 × g for 5 min to clear cell debris and aliquots stored at -80°C.

To determine the titer of SARS-CoV-2 virus stocks, VeroE6 cells were seeded at 3 × 10^4^ cells per well in 48-well plates. After 24h, adherent cell monolayers were challenged with serial 1 in 10 duplicate dilutions of virus and titer was assessed after 20h by in situ intracellular staining to identify foci of infection. Cells were washed in PBS, fixed in ice-cold methanol:acetone (50:50) and virus antigen was stained using sera from convalescent individuals diluted 1 in 2000 in PBS 1% FCS for 1h at 37°C. Cells were washed a further 3 times in PBS and incubated with goat anti-human IgG β-galactosidase-conjugated antibody (#2040-06, Southern Biotech) diluted 1 in 400 in PBS 1% FCS for 1h at 37°C. After 3 further PBS washes, 300μl of 0.5mg/ml 5-bromo-4-chloro-3-indolyl ß-D-galactopyranoside chromogenic substrate (X-gal) in PBS containing 3 mM potassium ferricyanide, 3 mM potassium ferrocyanide and 1 mM magnesium chloride was added to each well. Infected cells incubated at 37°C stained blue within 1 and 4h after addition of substrate and clusters of blue cells were counted as foci of infection to determine the virus titer defined as focus forming units (FFU) per ml.

### Authentic SARS-CoV-2 microneutralization assays

VeroE6 cells were seeded at 2 × 10^4^ cells per well in a clear, flat-bottom 96-well tissue culture plate 24h before infection. Participant serum was heat-inactivated for 30 min at 56°C to remove complement activity. Serum dilutions in DMEM were performed in duplicate in clear u-bottom 96 well plates with a starting dilution of 1 in 20 and 7 consecutive 2-fold dilutions to a final dilution of 1/2,560 in a total volume of 50μl per well. 3 × 10^4^ FFU of SARS-CoV-2 virus were added to each serum dilution and incubated at 37°C for 1h. After incubation, serum/virus preparations were transferred into cell culture plates containing semi-confluent VeroE6 monolayers. Each plate had 8 control wells with virus and cells only (virus control) and another 8 wells with cells only (background only). Plates were incubated (37°C and 5% CO_2_) for 72h, after which supernatants were removed and wells washed with PBS. Cells were fixed with 100 μl 3.7% (vol/vol) formaldehyde for 1h. After two further PBS washes, cells were stained with 0.2% (wt/vol) crystal violet solution for 10 min. Plates were washed four times in distilled water to remove excess crystal violet and left to air dry. Crystal violet stain was re-solubilized by addition of 100μl 1% (wt/vol) sodium dodecyl sulfate solution to each well and incubated at 37°C for 10 min. Absorbance readings were taken at 570nm using a CLARIOStar Plate Reader (BMG Labtech). Negative controls of pooled pre-pandemic sera, collected prior to 2008, and serum from a neutralization positive SARS-CoV-2 convalescent individual were spaced throughout the plates. Absorbance readings for each well were standardized against technical positive (virus control) and negative (cells only) controls on each plate to determine a percentage neutralization value. An average neutralization was calculated across the two sample replicates for each serum dilution. Neutralization curves for each serum tested were plotted, with the percentage neutralization modeled as a logistic function of the serum dilution factor (log10). A non-linear regression (curve fit) method was used to determine the dilution fold that neutralized 50% (IC50). We classified positive samples as those with an IC50 greater than 49. SARS-CoV-2 is classified as a hazard group 3 pathogen and therefore all authentic SARS-CoV-2 propagation and microneutralization assays were performed in a containment level 3 facility.

### Statistics and reproducibility

Data was assumed to have a non-Gaussian distribution. Nonparametric tests were used throughout. For single paired and unpaired comparisons Wilcoxon matched-pairs signed rank test and a Mann-Whitney U test were used. For multiple paired and unpaired comparisons Friedman multiple comparisons ANOVA with Dunn's correction or Kruskal-Wallis one-way ANOVA with Dunn’s correction were used. For correlations, Spearman’s r test was used. A p value <0.05 was considered significant. Prism v. 7.0e and 8.0 for Mac was used for analysis.

## SUPPLEMENTARY MATERIALS

immunology.sciencemag.org/cgi/content/full/5/54/eabf3698/DC1 Fig. S1. T cell responses to SARS-CoV-2 antigens in HCW with and without laboratory-confirmed SARS-CoV-2 infection 16-18 weeks after UK lockdown Fig. S2. SARS-CoV-2 specific immune responses in HCW with laboratory-confirmed infection by age Fig. S3. SARS-CoV-2 specific immune responses in HCW with laboratory-confirmed infection by gender and ethnicity Fig. S4. Correlation between IC50s measured using pseudovirus and authentic virus neutralization assays Fig. S5. Demographic characteristics of HCW with laboratory-confirmed SARS-CoV-2 infection but no nAb at 16-18 weeks after UK lockdown Fig. S6. Demographic characteristics of health care workers with laboratory-confirmed SARS-CoV-2 infection but no T cell response to Spike or N protein at 16-18 weeks after UK lockdown Fig. S7. Correlations between antibody and T cell responses in HCW Fig. S8. T cell responses to SARS-CoV-2 in HCW with laboratory-confirmed infection stratified by symptoms Fig. S9. S1 IgG antibody titers and neutralizing antibody at 16-18 weeks after the first UK lockdown Fig. S10. T cell and nAb responses similar across recruitment cohort 1 and 2 in the cross-sectional sub study 16-18 weeks after UK lockdown Table S1. HCW characteristics and COVID-19 status Table S2. Mapped epitope peptide (MEP) pools, spike MEP2 pool and overlapping peptides (OLP) pool. Table S3. Characteristics of pre-pandemic COVID-19 controls Table S4. Raw data file (Excel spreadsheet)
